# Heat-Inactivated Selenium Nanoparticle-Enriched *Lactobacillus* Enhance Mucosal IgA Responses and Systemic Responses of *Clostridium perfringens* Multi-Epitope Vaccine Correlated with TGF-β and NF-κB Pathways in Mice

**DOI:** 10.3390/microorganisms14010180

**Published:** 2026-01-14

**Authors:** Xinyao Zhou, Zheng Jia, Xinqi De, Zaixing Yang, Yifan Li, Runhang Liu, Lingdi Niu, Xinran Yao, Yuxuan Jiang, Fang Wang, Junwei Ge

**Affiliations:** 1 State Key Laboratory of Animal Disease Control and Prevention, Harbin Veterinary Research Institute, Chinese Academy of Agricultural Sciences, Harbin 150069, China; 2 Heilongjiang Provincial Key Laboratory of Zoonosis, College of Veterinary Medicine, Northeast Agricultural University, Harbin 150030, China; 3 Department of Veterinary Medicine, College of Agriculture, Liaodong University, Dandong 118003, China

**Keywords:** nano selenium, probiotics, multi-epitope vaccine, mucosal IgA, adjuvant

## Abstract

*Clostridium perfringens* is one of the main causes of death in poultry with no vaccines approved for poultry at present. The appropriate adjuvant is critical for the development of vaccines in *C. perfringens* in poultry. Here, we utilized *Levilactobacillus brevis* for high-yielding selenium biotransformation and demonstrated that heat-inactivated nano-selenium *Lactobacillus* (HiSeL) is a safe, efficient, and chemically stable selenium immunopotentiator for *C. perfringens* vaccines. We evaluated the effectiveness of HiSeL as an immune adjuvant to modulate the efficacy of multi-epitope vaccine in mice. Subcutaneous immunization mice with HiSeL promoted high levels of specific IgG, modulated cytokine secretion, downregulated stress-related gene expression, and provided 100% protection against lethal challenge with *C. perfringens*. Surprisingly, we found that HiSeL can quickly and effectively induce SIgA production even by subcutaneous immunization. Transcriptome sequencing revealed the pivotal role of TGF-β and NF-κB signaling pathways in IgA immune responses in mice immunized with the HiSeL-adjuvanted multi-epitope vaccine. Collectively, our study provides proof-of-concept evidence that HiSeL functions as a potent adjuvant candidate for the multi-epitope vaccine in a murine model, offering new insights into the development of engineered postbiotic-based adjuvants.

## 1. Introduction

Necrotic enteritis (NE), which is caused by *Clostridium perfringens*, is one of the most common enteric diseases in the poultry industry. It leads to enteric diseases with increased mortality, impaired production, and potential contamination of poultry products [1]. Currently, antibiotics have been prohibited in animal feed as growth promoters, resulting in a significant rise in necrotic enteritis outbreaks within the poultry industry. Vaccination is the most cost-effective method of disease prevention, but no commercial vaccines are approved for poultry [2]. Traditional vaccine development approaches depend on using whole, weakened, or dead microorganisms and inactivated bacterial toxins. However, these methods come with risks of potential reactivation or recombination of the vaccine strain, and they offer lower protective efficacy and immunity compared to newer technologies [3]. In recent years, there has been a growing interest in the use of multi-epitope vaccines, which consist of a novel protein linked to an immunogenic epitope. These multi-epitope vaccines offer several advantages in a broader spectrum of pathogen variation, eliciting cytotoxic T lymphocyte, helper T lymphocyte, and B cell responses, reducing adverse effects over conventional vaccines [4,5]. However, new vaccine formulations are required to stimulate well-defined cellular and humoral immune responses [6]. Therefore, in order to elicit robust immune responses, such as humoral, cellular, and mucosal immune responses, new immunopotentiators or immune-stimulating adjuvants are highly needed in vaccine preparations [7].

Selenium, a crucial mineral for the body, plays a significant role in preserving the equilibrium of the human immune system, endocrine system, metabolism, and intracellular environment [8,9]. The chemical composition of selenium heavily impacts its biological and toxicological effects. Nano-selenium in elemental form demonstrates lower toxicity and higher bioavailability than inorganic selenium, rendering it the preferred supplementary source of selenium [10]. Lactic acid bacteria (LAB) have been extensively studied for their potential applications in the bioconcentration and bioconversion of inorganic selenium into elemental and organic forms [11]. Selenium-rich probiotics are considered to be more effective organic selenium supplements. A large number of in vitro and in vivo studies have proven the beneficial effects of selenium-rich LAB on health, such as antibacterial, antioxidant, and metabolism regulation [12]. Zhao et al. found that administration of selenium-enriched *Bifidobacteria* has a relieving effect on diabetic mice, and the effect is better than sodium selenite [13]. Supplementing selenium-rich *Lactobacillus acidophilus* and *Saccharomyces cerevisiae* in piglet feed improved poor growth performance, and alleviated renal damage and oxidative stress caused by ochratoxin A in pigs [14]. In a study to evaluate the ability of selenium-enriched probiotics to reduce oxidative stress, it was found that selenium-enriched *Bacillus paralicheniformis* was able to attenuate H_2_O_2_-induced apoptosis and oxidative damage in porcine jejunal epithelial cells through the MAPK pathway [15].

Studies have proven that selenium-rich probiotics have immune-modulating effects. A study by Huang et al. found that selenium and probiotics may have a synergistic effect in immune regulation. Adding selenium-rich *L. acidophilus* and *S. cerevisiae* to piglet feed can improve the antioxidant capacity of piglets (characterized by increased GPX, SOD activity and GSH content increase, and MDA content decreases), while T lymphocyte proliferation and serum IL-2 levels increase [16]. Moreover, the oral administration of nano-selenium *Lactobacillus plantarum* can effectively enhance the immune response and prevent breast cancer [17]. In addition, selenium-enriched *L. plantarum* and Bifidobacterium longum can act as immune modulators and reduce colon inflammation caused by piroxicam medication [18]. Therefore, selenium-rich probiotics have great potential as vaccine adjuvants to modulate immune responses. However, existing research mainly focuses on improving the production performance of farm animals with selenium-rich probiotics. There is almost no research on their use as vaccine adjuvants, and the immunomodulatory mechanism of selenium-rich probiotics is still unclear.

In our previous study, HiSeL’s potent induction of SIgA production compensated for the lack of mucosal immunity induced by alum and provided protection against a lethal challenge with *C. perfringens* type A [19]. In this study, we aimed to improve the multi-epitope vaccine (CPMEA-OACD-BLP23017) for chicken necrotizing enteritis, which was previously developed in our laboratory. Subcutaneous injection of HiSeL promoted high levels of specific IgG, modulated cytokine secretion, reduced stress-related gene expression, and provided 100% protection against lethal challenge with *C. perfringens*. We demonstrated that HiSeL has the capacity to trigger the generation of SIgA by activating the TGF-β and NF-κB signaling pathways, consequently bolstering the immune reaction induced by the multi-epitope vaccine. Hence, HiSeL enhances the efficacy of the multi-epitope vaccine against *C. perfringens*, demonstrating its strong potential as a potent mucosal adjuvant candidate.

## 2. Materials and Methods

### 2.1. Optimized Preparation of HiSeL

HiSeL was prepared by thermal inactivation of SeL as previously described [20]. SeL was obtained under a biosynthetic system with sodium selenite as the selenium source. In order to explore the optimal preparation conditions, we added different concentrations of sodium selenite (5–45 μg/mL) at different bacterial culture time points (0, 4, 9 and 14 h). The culture was then continued for 18 h at 37 °C.

We used the 3,3′-diaminobenzidine colorimetric method [21] to measure the selenium enrichment ability of LAB to evaluate the conversion rate of SeNPs. We used toluene to extract the inorganic selenium in the final culture supernatant and measured its OD_420nm_. The inorganic selenium content was obtained by the standard curve (y = 0.0039x + 0.0013, R^2^ = 0.9926). Selenium enrichment capacity (conversion of selenium) = (total selenium content − residual inorganic selenium content)/total selenium content × 100%.

Purification of selenium nanoparticles was carried out as described earlier [22]. In brief, SeL is disrupted using ultrasonic waves, and SeNPs are extracted from the resulting sediment using secondary octanol. The SeNPs are then meticulously washed and purified using chloroform, ethanol, and sterile water. The obtained SeNPs are freeze-dried for FTIR.

### 2.2. Characterization of HiSeL

The HiSeL composites were characterized by a series of advanced techniques. Dynamic light scattering (DLS) was utilized to measure the particle size and zeta potential using a particle size analyzer (Malvern, UK). Transmission electron microscopy (TEM, H-7650; Hitachi, Ltd., Tokyo, Japan) was used to determine the morphological characteristics of HiSeL. HiSeL suspended in glutaraldehyde were added to carbon-coated copper TEM grids, and samples were dried at RT before observation. The samples were stained with phosphotungstic acid. The FTIR spectra (ThermoFisher Scientific, Waltham, MA, USA) of SeL and HiSeL were mixed with dried KBr powder and detected in the range of 400–4000 cm^−1^.

The particle-based dose of HiSeL per dose is measured using Microfluidic Resistive Pulse Sensing (MRPS) technology [23]. MRPS measurements are performed using the nCS1 instrument (Spectradyne, Signal Hill, CA, USA). The selenium (Se) content of HiSeL was determined by inductively coupled plasma mass spectrometry (ICP-MS), following previously described protocols [24]. Briefly, 1 mL of HiSeL was added to a mixture of 1.6 mL of nitric acid (65%) and 1.6 mL of hydrogen peroxide (40%) at 200 °C until dry. The residue underwent sequential acid digestion with 0.56 mL HCl at 100 °C and 0.8 mL H_2_SO_4_ (50 °C to 130 °C) until complete dryness. Finally, the samples were diluted in 20 mL of distilled water, filtered through a 0.22 μm membrane, and analyzed using an ICP-MS system (Agilent Technologies, Santa Clara, CA, USA).

### 2.3. Preparation and Identification of Multi-Epitope Vaccine

CPMEA-OACD-BLP23017 (multi-epitope vaccine) was prepared according to the method established in our laboratory [25]. Briefly, we used CPMEA as the target antigen, which is an epitope group of key antigens responsible for *C. perfringens* infection, leading to necrotizing enteritis in chickens. *E. coli* Rosetta containing a recombinant plasmid was cultured overnight in a resistance medium. To induce the expression of the fusion protein, 0.5 mM of IPTG was added, and the culture was grown overnight at 22 °C with shaking. Then, the soluble protein was purified according to the instructions of a His-tag purification resin (in denaturing agent form) kit and a bacterium-like particle (BLP) surface display system was used as carrier. BLPs are peptidoglycan skeletons that remain in lactic acid bacteria (LAB) after hot acid treatment, retaining the shape and size of LAB without any active cellular components. We predicted the functional domains of the C-terminal domain of OmpA (named OACD) through bioinformatics analysis and used OACD as an anchor to bind to BLP23017 made from *Levilactobacillus brevis* 23017 to construct the OACD-BLP23017 surface display system [26]. We have established a BLP, displaying multi-epitope vaccine and confirming its immune-enhancing effect. The CPMEA displaying on BLP were analyzed by SDS-PAGE, Western blot, and immunofluorescence which were all conducted following standard methods.

The experiment detected the binding of *Clostridium perfringens* type A-positive serum to BLPs (bacterial-like particles) using immunofluorescence. First, 200 µL of the binding product and protein-free BLPs (negative control) were spotted onto glass slides and air-dried. The samples were then blocked with 5% skim milk for 1 h, followed by three washes with PBST. Next, the samples were incubated with a 1:1000 dilution of the primary antibody (positive serum) for 45 min, washed, treated with a 1:2000 dilution of FITC-labeled goat anti-rabbit IgG (secondary antibody) in the dark for 45 min, and then put through additional washes. Finally, the slides were mounted with antifade mounting medium, sealed with nail polish, stored in the dark, and observed under a fluorescence microscope.

### 2.4. Immunization and Challenge

All female Kunming mice (6–8 weeks old) were purchased from Changsheng Biological Company (Liaoning, China). All animal studies and housing facilities were approved by the Institutional Animal Care and Use Committee of Northeast Agricultural University under permit number NEAUEC20210326.

Forty-eight female Kunming were randomly divided into four groups equally (n = 12). The mice were vaccinated subcutaneously with multi-epitope vaccine in the Vac group (50 μg antigen), with multi-epitope vaccine (50 μg of antigen) and Alum (50 μL) in the Vac + AL group, with multi-epitope vaccine (50 μg of antigen) and HiSeL (1.5 × 10^9^ particles) in the Vac + HiSeL group, and with PBS (0.2 mL) in the Con and infection group, at a total volume of 0.2 mL for all of the samples. Six mice from each group were intraperitoneally administered a lethal dose of *C. perfringens* (10^8^ CFU) at either 6 or 21 dpi. Survival rates and body weight loss were monitored daily for 7 dpc, and tissue samples, blood and stool were harvested from the groups of mice at 7 dpc. The allocation within each group was as follows: Six mice were challenged at 6 dpi to monitor survival rates. The remaining six mice were challenged at 21 dpi. Serum and fecal samples were collected on days 5, 14, 21, and 28 for IgA detection. Serum samples from these time points were also analyzed for IgG titers, cytokine expression (TGF-β1 and IL-5), and toxin neutralization capacity. On day 28, spleens were harvested for RT-qPCR and histopathological examination.

For RNA-Seq, nine female Kunming mice were randomly divided into three groups equally (n = 3). Mice were injected subcutaneously with Vac or Vac + HiSeL (at the equivalent dosage of 50 μg antigens and 1.5 × 10^9^ particles HiSeL per mouse). At 3 dpi, the spleens of mice were collected for transcriptomic analysis. Three samples were prepared for each group.

### 2.5. Antibody Measurement by ELISA

For detection of specific IgG, IgG1, IgG2a, and IgA antibodies, serum and stool samples were collected and at 5, 14, 21, 28 dpi. The ELISA plates were coated with 3 μg/mL antigen overnight at 4 °C, washed five times with PBST, and blocked with 2% BSA in PBS for 2 h at 37 °C. After another wash, 100 μL of diluted samples were added and incubated for 1.5 h at 37 °C. Following further washing, HRP-labeled antibodies were added for 1 h at 37 °C. After final washes, the reaction was developed with TMB substrate for 30 min at 37 °C and stopped with Stop Solution; absorbance was measured at 450 nm. Antibody titers were determined as the highest serum dilution, yielding OD450 > 0.2.

For fecal sample processing, samples were weighed and homogenized in 0.05 mol/L EDTA-Na_2_-PBS solution. The volume of the buffer was determined based on fecal weight using the following formula: Volume (μL) = Fecal Weight (g) × 4000 (e.g., 400 μL of solution per 0.1 g of feces). The homogenate was then centrifuged at 12,000× *g* for 10 min at 4 °C to remove particulate matter and obtain a clear supernatant. The supernatant was kept frozen until use. Specific antibodies were detected by indirect ELISA according to Krähling et al. [27] with some modifications. In the case of measuring high-affinity antibodies, a modified ELISA protocol with urea washes was used. Plates were coated with 1.5 µg purified antigen protein per well overnight at 4 °C and blocked with 5% skim milk. Appropriate dilutions of the samples were hatched for 1 h at 37 °C. Peroxidase-conjugated anti-mouse antibodies for IgG, IgG1, IgG2a, and IgA were added and incubated for 1 h at 37 °C.

Considering that the intestine secretes mucus to form a mucus barrier, we collected intestinal mucus to detect total intestinal mucus SIgA. A 5 cm segment of the distal ileum was excised and flushed with 1 mL of sterile phosphate-buffered saline (PBS, pH 7.2). The intestinal lavage fluid was centrifuged at 12,000× *g* for 10 min at 4 °C. The supernatant was collected for subsequent sIgA antibody detection. SIgA antibody assay was performed according to the instructions of a commercial ELISA kit (Sangon Biotech, Shanghai, China).

### 2.6. Cytokine Assays

TGF-β1 and IL-5 in serum were tested by ELISA kits (Boster Bioengineering, Wuhan, China) following the manufacturer’s protocol.

RT-qPCR was used to analyze the expression of *IL-4*, *IL-10*, *IL-12*, *IFN-γ*, *IL-1β*, *TNF-α*, *Star*, *P450scc*, *Hsp70*, *Hsp90*, *TGF-β1*, *Cxcl12*, *Map3k14*, *pIgR*, *J chain* and *IκBα* in spleen and Payer’s patches. The primers are listed in Supplementary Table S1. A volume of 5 μg of total RNA was extracted from spleens and Ilium Payer’s patches using the TRIzol reagent (Invitrogen, Waltham, CA, USA) and reverse-transcribed using ReverTra Ace (Toyobo, Osaka, Japan). RT-qPCR was performed using 2 × SYBR green master mix (Biotool) in a 7500 real-time system (Applied Biosystems, Foster City, CA, USA) and the data are presented as the mRNA accumulation index (2^−ΔΔCt^).

### 2.7. Histopathology

To evaluate intestinal histological changes, ileums were obtained at 7 dpc from mice. Ileum samples were fixed in 10% formalin and the sectioning and haematoxylin–eosin (HE) staining were entrusted to Seville Biotechnology (Wuhan, China). Finally, the images were captured using a microscope (Nikon Eclipse E100, Tokyo, Japan).

### 2.8. Toxin Neutralization Assay

The in vitro toxin-neutralizing capacity of serum from immunized mice was evaluated according to a previously described method [28]. Briefly, the culture supernatant of the challenge strain, *C. perfringens* C57-8, was mixed with an equal volume of serum from immunized mice and incubated at 37 °C for 2 h. Subsequently, 10 µL of the mixture was spotted onto blood agar plates. The plates were incubated overnight, and the hemolysis zones were observed the following day.

### 2.9. RNA-Seq Analysis

The RNA-Seq analysis was entrusted to the BGI (Shenzhen, China). In brief, mouse spleen total RNA was extracted using Trizol Reagent (Thermo Fisher Scientific, Waltham, MA, USA). Library construction and sequencing were performed by on the DNBSEQ platform. Essentially, differential expression analysis was performed using the DESeq2 (v1.4.5) with |log_2_ (fold change)| > 1 and *p* value ≤ 0.05.

All DEGs were further analyzed by GO (http://www.geneontology.org/ (accessed on 1 January 2023)) and KEGG (https://www.kegg.jp/ (accessed on 1 January 2023)) enrichment with a *p* value ≤ 0.05. We performed the GSEA by using GSEA v3.0 (http://www.broad.mit.edu/gsea/ (accessed on 1 January 2023)). The STRING database (https://www.string-db.org/ (accessed on 1 January 2023)) was used to build the PPI networks for the identified DEGs to predict their interactions and a combination score of >0.5 was used as the threshold. Hub genes were selected by considering the high degree of connectivity in the PPI networks analyzed by the cytohubba plugin of Cytoscape 3.9.1.

### 2.10. Statistical Analysis

Each mouse was considered an individual biological replicate, and each experiment was repeated three times technically. All statistics were performed using GraphPad Prism 8.0.1 (GraphPad Software, San Diego, CA, USA). Quantitative data are reported as mean ± SD. *p* values were determined by one-way ANOVA analysis or two-way ANOVA and corrected for multiple comparisons using Tukey testing. Statistical significance was determined at *p* < 0.05 (*), *p* < 0.01 (**), and *p* < 0.001 (***).

## 3. Results

### 3.1. Optimized Preparation and Characterization of HiSeL

**Figure 1:**
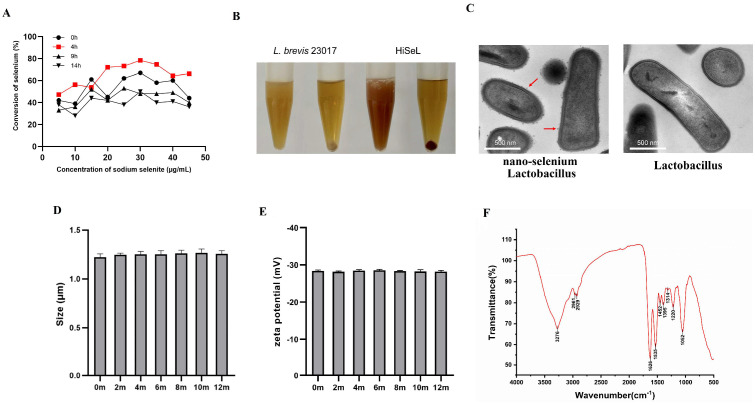
Characterization of HiSeL. (**A**) The effect of addition time and concentration of sodium selenite on the conversion rate of nano-selenium. (**B**) The optimized preparation of thermally inactivated nano-selenium *L. brevis* cell bodies is red. (**C**) TEM image of SeL with nano-selenium on the surface of the cell bodies (red arrows). (**D**) The average diameter and (**E**) zeta potential of HiSeL after storing at room temperature for 12 months. (**F**) FTIR spectrum of SeNPs synthesized by *L. brevis* 23017.

As shown in Figure 1A, the concentration and addition time of sodium selenite both affect the selenium conversion rate. When 30 μg/mL of sodium selenite was introduced during a 4 h culture of *L. brevis*, the selenium conversion rate reached its peak at 78.4%. The SeL bacterial liquid obtained after cultivation appeared red, and the precipitate after centrifugation reached a “full red” state (Figure 1B). The transmission electron microscope (TEM) image shows the structure of nano-selenium *Lactobacillus* (SeL), with a large number of selenium nanoparticles (SeNPs), approximately 50–80 nm in diameter, attached to the bacteria cell surface (Figure 1C). Stability results showed that HiSeL remained stable within 12 months when kept at room temperature with almost constant particle size and zeta potential (Figure 1D,E).

Additionally, the Fourier transform infrared (FTIR) spectra of the purified SeNPs from *L. brevis* are shown in Figure 1F. The absorption band at 3276 cm^−1^ is attributed to the stretching vibrations of the hydroxyl groups. The bands at 2961 cm^−1^ and 2929 cm^−1^ refer to the different stretched C-H vibrations of aliphatic groups, such as -CH_2_- and -CH_3_ (which are present in protein side chains and lipids) [29]. In the low wavenumber region, 1626 cm^−1^ is assigned to the carbonyl C=O stretch, 1535 cm^−1^ to amide I vibration, 1452 cm^−1^ to amide II, and 1220 cm^−1^ to amide III vibrations. The peak at 1395 cm^−1^ may be due to the O–H bending of carboxylate. The most intense vibrational band at 1052 cm^−1^ corresponded to characteristic Se–O bond stretching, according to previously reported data in the literature [30]. In summary, the FTIR analysis indicated the presence of organic residues such as carbohydrates, proteins, and lipids on the surface of the SeNPs produced by *L. brevis* 23017. Additionally, the particle concentration and selenium (Se) content per dose of HiSeL were quantified. Microfluidic Resistive Pulse Sensing (MRPS) and inductively coupled plasma mass spectrometry (ICP-MS) analyses revealed that each dose (200 μL) contained 1.5 × 10^9^ particles/200 μL and 0.1 µg of selenium.

### 3.2. Preparation of Multi-Epitope Vaccine

**Figure 2:**
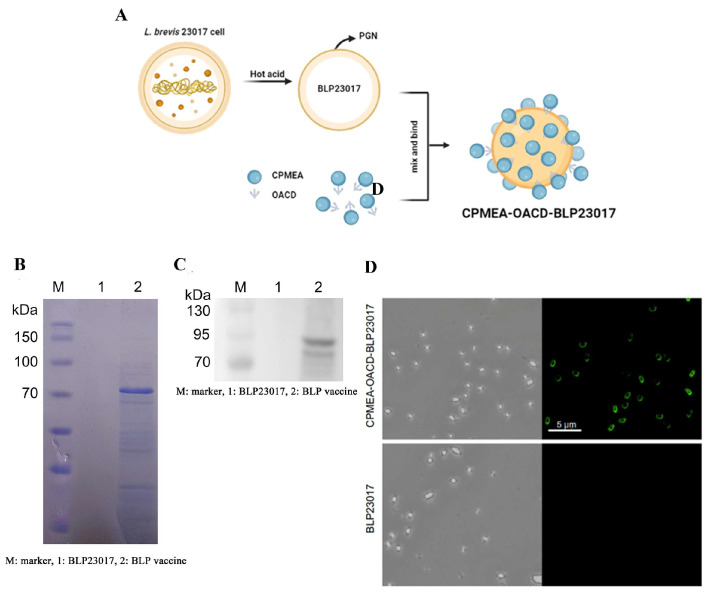
Preparation and identification of multi-epitope vaccines. (**A**) Illustration of the synthesis of bacterium-like particles (BLPs) displaying the multi-epitope vaccine. (**B**) SDS-PAGE of BLP displaying the multi-epitope vaccine. M: marker, 1: BLP23017, 2: BLP vaccine. (**C**) Western blot of BLP displaying the multi-epitope vaccine. M: marker, 1: BLP23017, 2: BLP vaccine. (**D**) Immunofluorescence assay detection of BLP displaying the multi-epitope vaccine.

The production process for the BLP-based multi-epitope vaccine is shown in Figure 2A, in which the CPMEA antigen is displayed at high density on the surface of BLP23017. Compared with the BLP23017, CPMEA-OACD (85 kDa) was shown to successfully bind to BLP after scanning of the SDS-PAGE gel and Western blot (Figure 2B,C). Meanwhile, bright green fluorescence was detected on the surface of BLP23017 by immunofluorescence analysis (Figure 2D). These results indicated that the CPMEA antigen could be successfully combined with BLP, and the multi-epitope vaccine was successfully prepared.

### 3.3. HiSeL-Enhanced Antibody Responses Induced by Multi-Epitope Vaccines

**Figure 3:**
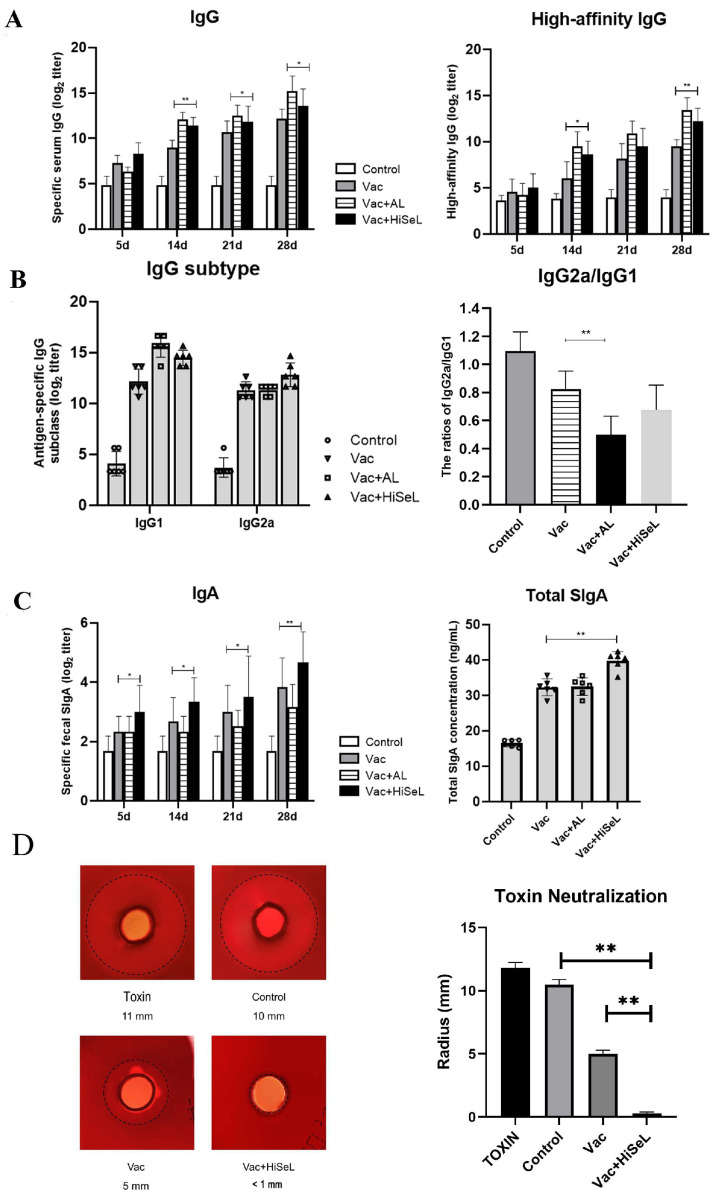
The specific antibody levels in immunized mice. (**A**) The serum-specific IgG and high-affinity IgG, (**B**) specific IgG subclasses, (**C**) specific fecal IgA and total intestinal mucosal SIgA levels. (**D**) Results of in vitro toxin neutralization experiment with mouse serum. Data are presented as mean ± SD (* *p* < 0.05, ** *p* < 0.01).

To evaluate the impact of HiSeL on the immune response, mice were subcutaneously immunized with the HiSeL-adjuvanted multi-epitope vaccine, using Alum adjuvant as a positive control. At 5 days post-immunization (dpi), HiSeL showed a slight increase in serum IgG titer, though there was no statistical difference. At this time, Alum adjuvant had not yet produced antibodies (Figure 3A). As time went by, the antibody levels in each group gradually increased and reached the highest level at 28 dpi. Starting from 14 dpi, HiSeL significantly increased vaccine-induced IgG titers compared to the Vac group (*p* < 0.05), but the effect was lower than those induced by the aluminum gel adjuvant. The generation of high-affinity antibodies is fundamental to neutralizing a broad range of pathogens. The results demonstrated that HiSeL can significantly increase high-affinity IgG levels on 28 dpi compared to single vaccine (*p* < 0.01).

To examine the type of humoral immune responses, IgG subclasses were analyzed (Figure 3B). HiSeL increased antibody titers of IgG subclasses and slightly decreased the IgG2a/IgG1 ratio from 0.82 to 0.68. To evaluate whether HiSeL can enhance mucosal immune responses, we measured specific IgA in feces and total IgA in the intestinal mucus of mice on 28 dpi (Figure 3C). IgA levels in feces and intestinal mucus were significantly higher in the Vac + HiSeL group compared to the Vac group (*p* < 0.01). These results confirm the adjuvant effect of HiSeL. Based on the IgG subclass ratios, HiSeL appears to induce an immune response characterized by elevated IgG1 levels, suggesting a profile that may differ from the classical Th2 dominance of the aluminum gel adjuvant. Notably, HiSeL shows outstanding advantages in inducing mucosal immunity. As shown in Figure 3D, the hemolysis zones in the Control group were similar in size to those of the α-toxin positive control, indicating that serum from the Control group possessed negligible toxin-neutralizing activity. In contrast, the hemolysis zones in the Vac group were significantly smaller than those in the Control group. Notably, the hemolysis zones in the Vac + HiSeL group were barely visible. These results demonstrate that Vac + HiSeL immunization induced the most potent production of toxin-neutralizing antibodies.

### 3.4. HiSeL Regulate the Expression of Cytokine

Next, to assess T cell responses, we examined cytokines induced in the mouse serum and spleen in response to immunization at 28 dpi. TGF-β and IL-5 expressions in serum were measured in ELISA assays. IL-4, IL-10, IL-12, IFN-γ, IL-1β, TNF-α expressions in spleens were measured by real-time quantitative PCR (RT-qPCR) assays.

**Figure 4:**
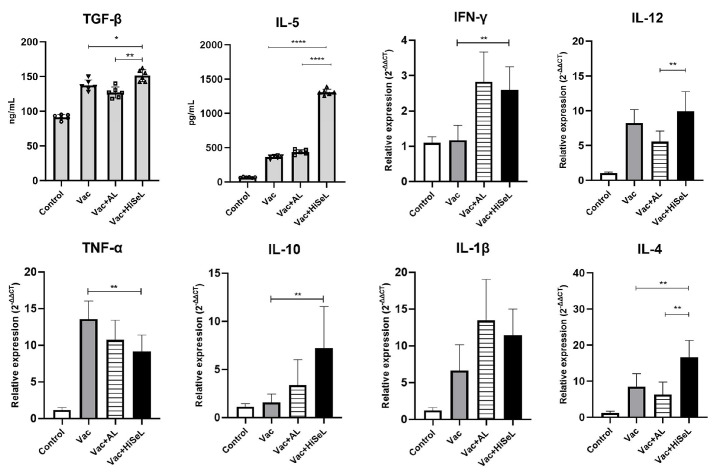
The cytokines induced by HiSeL in mice. The levels of TGF-β and IL-5 were measured at 28 dpi in the serum using ELISA, and the mRNA expression levels of IFN-γ, IL-12, TNF-α, IL-10, IL-1β, and IL-4 in the spleen were measured by RT-qPCR. The data are presented as mean ± SD (* *p* < 0.05, ** *p* < 0.01, **** *p* < 0.0001).

TGF-β and IL-5 are important immunoregulatory cytokines and are closely related to SIgA secretion. As shown in Figure 4, on the 28 dpi, TGF-β (*p* < 0.05) and IL-5 (*p* < 0.001) secretion by spleens from mice immunized with Vac + HiSeL was significantly increased compared to the Vac and Vac + AL groups. The mRNA expression of IFN-γ, IL-4, and IL-10 in the Vac + HiSeL group (*p* < 0.01) was significantly increased compared with the Vac group, indicating that both the Th1 and Th2 arms of adaptive immunity were activated. We also observed increased IL-12 and IL-1β levels in the Vac + HiSeL group, but there was no significant difference. Compared to the Vac group, TNF-α expression was decreased in the Vac + HiSeL group (*p*  <  0.01). The levels of pro-inflammatory cytokines, for both IL-1β and IFN-γ, were the highest for the Vac + AL group followed by the Vac + HiSeL group, indicating that aluminum adjuvant induced a more potent inflammatory response. Altogether, HiSeL induced significant T cell responses and cytokine expression that may favor SIgA production.

### 3.5. HiSeL Defense Against Lethal C. Perfringens Challenge

**Figure 5:**
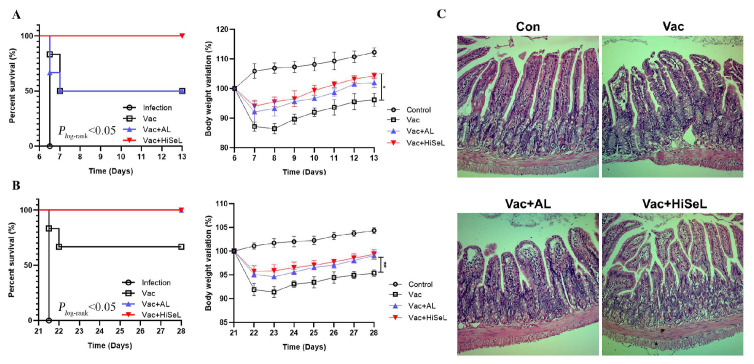
Survival curve and weight change in mice after challenge at (**A**) 6 dpi and (**B**) 21 dpi. (**C**) Histopathological evaluation of the ileum at 28 dpi (7 dpc). The data are presented as mean ± SD (* *p* < 0.05, ** *p* < 0.01).

To further determine the ability of HiSeL to provide protection, we challenged mice intraperitoneally with *C. perfringens* type A C57 on 6 dpi (Figure 5A) and 21 dpi (Figure 5A), respectively, and monitored survival and body weight changes for 7 days post challenge (dpc). In both challenge experiments, all control mice died within 1 dpc. In the challenge experiment of 6 dpi, the survival rate for mice in the Vac group and the Vac + AL groups was 50%, while the survival rate of the Vac + HiSeL group achieved a remarkable 100% survival rate. Similarly, in the challenge experiment of 21 dpi, the survival rate for mice in the Vac group was 66.7%. The Vac + HiSeL group and Vac + AL group mice had a survival rate of 100%, underscoring the potent infection control and protective efficacy of HiSeL.

As shown in Figure 5A, on the first day after challenge (7 dpi), mice in the Vac + AL (7.9% of initial weight on average) and Vac + HiSeL groups (5.9%) showed less weight loss than the vaccine group (12.8%). The mice in the Vac + HiSeL group and the Vac + AL group showed similar performance in weight recovery after challenge. On the second day after the challenge (8 dpi), the body weights of mice began to recover, and reached 104.3% (Vac + HiSeL) and 102% (Vac + AL) of the initial body weight, respectively, on the seventh day after the challenge (13 dpi), which were significantly higher than the Vac group (96.2%) (*p* < 0.05). Similar weight changes in mice were seen after the 21 dpi challenge (Figure 5B). The weight of mice (99.3%) in the group receiving the HiSeL-adjuvanted vaccine was significantly increased compared to the Vac group (95.3%) (*p* < 0.01).

Sections of ileum in each group are shown in Figure 5C. Compared with the Vac group, mucosal inflammatory cell infiltration, separation of lamina propria and mucosal epithelium caused by loss of mucosal base and mucosal edema in the Vac + HiSeL group were significantly improved. Taken together, these results indicate that HiSeL protected mice against lethal *C. perfringens* challenge.

### 3.6. HiSeL Reduces mRNA Levels of Stress Gene Induced by Vaccination

**Figure 6:**
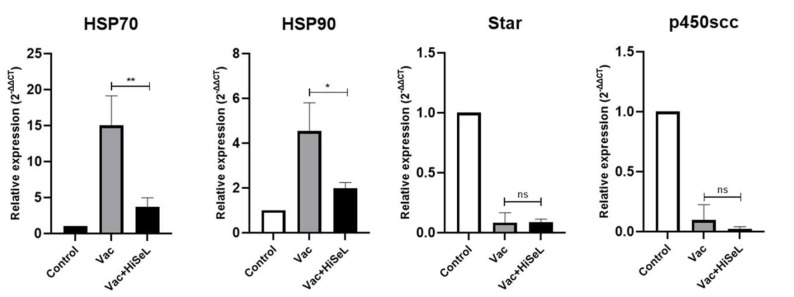
The expression of stress genes in the spleen. The expression of HSP70, HSP90, Star, and p450scc in the spleen was determined by RT-qPCR at 3 dpi. The data are presented as mean ± SD (* *p* < 0.05, ** *p* < 0.01, ns, not significant).

Three days after immunization, the mRNA levels of stress-related genes in the spleens in each group are shown in Figure 6. Compared to the Vac group, the levels of heat shock-related genes HSP70 (*p* < 0.01) and HSP90 (*p* < 0.05) were significantly reduced in the Vac + HiSeL group. There was a significant down-regulation of the transcription of key enzymes for cortisol production (StAR and P450scc) compared to control group.

### 3.7. HiSeL-Adjuvant Vaccine Causes Gene Expression Differences

**Figure 7:**
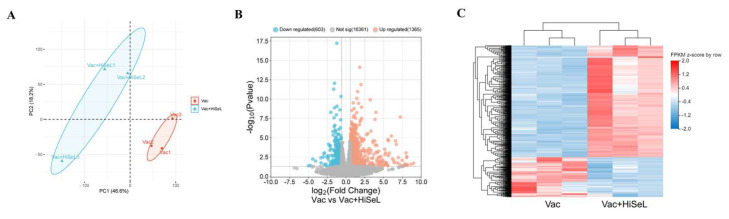
Transcriptome analysis of spleen in mice. The DEGs are identified by two criteria: (1) |log_2_ (fold change)| > 1; (2) *p* < 0.05. (**A**) PCA, (**B**) volcano plot, and (**C**) heatmap of DEGs between the Vac + HiSeL group and the Vac group.

The effect of immune enhancement induced by HiSeL was investigated using RNA-seq analysis. Principal Component Analysis (PCA) results showed a good separation of samples of Vac and Vac + HiSeL samples (Figure 7A). Using DESeq2, we identified 1968 differentially expressed genes (DEGs), including 1365 upregulated and 603 downregulated genes in the Vac + HiSeL group relative to the Vac group (Figure 7B). Heatmap analysis showed that samples in the Vac + HiSeL group showed distinct gene expression profiles from those of the Vac group (Figure 7C).

Since selenium mainly exerts its biological functions through selenoproteins and selenoenzymes [31], we analyzed the expression of selenoprotein and selenoenzyme genes. The heat map results showed that HiSeL can regulate the transcription levels of selenoprotein and antioxidant enzyme genes (Supplementary Figure S1). These differentially expressed genes include upregulated genes, namely selenot, selenoh, selenok, selenoo, gpx3, selenow, sod3, gpx7, and downregulated genes, namely sod1, gpx1, gpx4, dio1, selenoi, selenof, sephs2, sod2. In short, HiSeL can function in immunized mice and cause differential expression of genes.

### 3.8. HiSeL Activates Immune-Related Pathways

**Figure 8:**
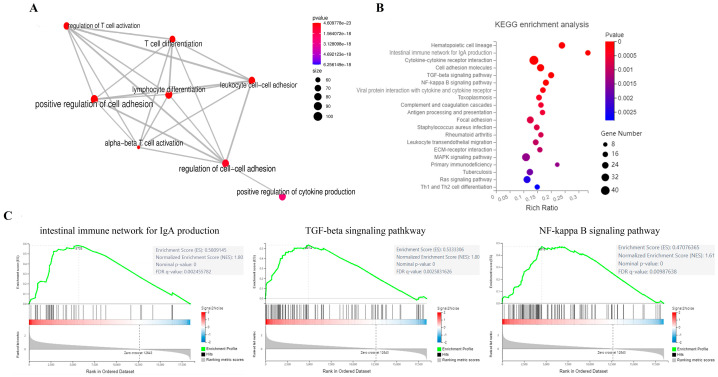
Enrichment analysis of (**A**) GO (biological process) and (**B**) KEGG for the identified DEGs in the spleen of mice. (**C**) The intestinal immune network for IgA production, NF-κB, and TGF-β signaling pathways was notably enriched in GSEA (NES > 0 represents pathway activation, while NES < 0 represents pathway suppression).

GO and KEGG analysis showed that DEGs are closely related to intestinal immunity. The top eight biological process (BP) terms significantly enriched in GO analysis are all related to the activation and proliferation of lymphocytes (Figure 8A). Figure 8B illustrates the KEGG pathways, with a focus on immune-related pathways such as hematopoietic cell lineage, intestinal immune network for IgA production, cytokine–cytokine receptor interaction, and others, including TGF-β signaling pathway, NF-κB signaling pathway, antigen processing and presentation, leukocyte transendothelial migration, MAPK signaling pathway, Ras signal pathway, and Th1 and Th2 cell differentiation.

Given the pronounced mucosal immune response observed during our investigation into HiSeL’s immunoenhancement, we delved deeper into the mechanisms underlying the intestinal immune network for IgA production, TGF-β signaling pathway, and NF-κB signaling pathway. The gene set enrichment analysis (GSEA) results showed that the TGF-β signaling pathway, intestinal immune network for IgA production, and NF-κB signaling pathway are significantly enriched and are activated by HiSeL, which verified the results obtained by KEGG (Figure 8C).

### 3.9. NF-κB and TGF-β Signaling Pathways Are Related to IgA Production Induced by HiSeL

**Figure 9:**
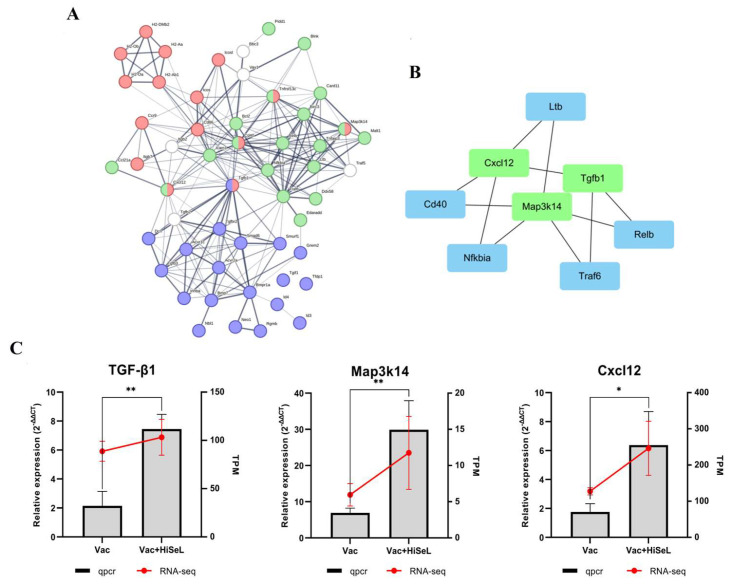
(**A**) PPI network of DEGs in enriched pathway (red: the intestinal immune network for IgA production pathway, green: the NF-κB signaling pathway, and purple: the TGF-β signaling pathway, and white: genes with high connectivity between pathways but no significant differential expression). (**B**) Hub genes (*Tgfb1*, *Cxcl12*, and *Map3k14*) in the PPI networks. (**C**) Comparison of the expression of RT-qPCR and RNA-seq’s TPM for *Tgfb1*, *Cxcl12*, and *Map3k14*. The bar graphs represent RT-qPCR detection of gene expression in mice spleens (2^−ΔΔCt^) and the red lines indicate the gene (TPM) in RNA-Seq. All genes in the figure show significant differences in the RNA-seq results. The significant labeling in the figure (*: *p* < 0.05, **: *p* < 0.01) based on RT-qPCR test results.

To investigate the potential relationship between IgA production and the NF-κB and TGF-β signaling pathways, we imported the DEGs into the search tool for the Retrieval of Interacting Genes (STRING) website for protein–protein interaction (PPI) analysis. PPI network analysis demonstrated the close relationship of proteins encoded by these genes (Figure 9A). Subsequently, we employed cytoHubba to identify and rank the top three hub genes, which were *Tgfb1*, *Cxcl12*, and *Map3k14*, as illustrated in Figure 9B. To validate the RNA-seq results, we performed RT-qPCR to assess the mRNA expression levels of these hub genes. As shown in Figure 9C, the mRNA level of Tgfb1, *Cxcl12*, and *Map3k14* were upregulated compared to the Vac group, consistent with the findings from RNA-seq. Furthermore, we extended our analysis to include the expression of some important genes (J Chain, IκB and PIgR) in Peyer’s patches and spleen to corroborate the RNA-seq results (Supplementary Figure S2). The overall trend remained largely consistent, further affirming the reliability of the RNA-seq findings. These results show that the NF-κB and TGF-β signaling pathways are related to IgA production induced by HiSeL.

## 4. Discussion

In recent decades, numerous studies have shown that selenium supplements have multiple outstanding biochemical properties, including antitumor, antioxidative, antiviral, immunomodulatory, and anti-atherosclerotic activities. The role of selenium as an adjuvant can induce good protective immune responses in vaccine components [32], and in the presence of selenium deficiency, innate and adaptive immune responses are impaired [33]. As biological response modifiers, most LAB can activate the immune system, and their potential as adjuvants has been previously reported, including *L. brevis* [2]. Previous studies in our laboratory found that inactivated selenium-enriched *L. brevis* can enhance the immune effect of aluminum-adjuvanted inactivated *C. perfringens* type A vaccine in mice [19]. However, whether HiSeL can improve the immune effect of multi-epitope vaccines needs to be evaluated. This study optimized the preparation of HiSeL and used mouse models to evaluate the immune-enhancing effect of HiSeL on multi-epitope vaccines and to explore its mechanism of action.

Probiotics or yeast can convert sodium selenite into organic selenium through biotransformation, but the reduction in sodium selenite by LAB has the disadvantages of low conversion rate, small yield, uneven particle size, and complicated and harsh preparation conditions [11]. In this experiment, the probiotic *L. brevis* 23017, which was isolated in the previous laboratory for antibacterial, anti-oxidation, anti-inflammation, and acid and alkali resistance, bile salt resistance, and pancreatin resistance, was selected to optimize the preparation of nano-selenium LAB. Our study identified the optimal preparation conditions for SeL by evaluating the effects of bacterial growth stage and sodium selenite concentration on selenium conversion rate. By measuring the growth curves of *L. brevis* 23017, we found that the isolate reached its log phase at 4 h, mid-log phase at 6 h and late-log phase at 14 h of incubation. Our results show that adding sodium selenite during the log phase (4 h) of the bacteria achieves higher conversion rates. A previous study found that *E. durans* LAB18s displayed the highest bioaccumulation of Se (IV) after 6 h of incubation, which is consistent with our results [34]. However, Chen et al. obtained the maximum strain selenium content and selenium conversion rate by adding sodium selenite at the early logarithmic stage (4 h) of bacterial growth [35]. Therefore, we speculate that the optimal growth stage for sodium selenite addition is strain-specific. Adding sodium selenite within a certain concentration can improve the selenium conversion rate, but too high a concentration of sodium selenite will inhibit the growth of the strain [35]. Our research found that the optimal sodium selenite concentration was 30 μg/mL, in which case the bacteria reached a “full red” form. The size of SeNPs is related to its biological activity [36]. It has been reported that smaller-sized particles of SeNPs have better scavenging effects on the free radicals [37]. The nano-selenium we produce has smaller particle size and does not have the problem of uneven particle size that is common with existing biologically converted nano-selenium [38].

More and more studies have found that inactivated probiotics contain the same cellular components and metabolites as live bacteria and have probiotic effects [39]. In 2019, the International Probiotics and Prebiotics Science Association classified inactivated probiotics as a type of postbiotics, defined as inanimate microorganisms that are beneficial to the health of the host [40]. Compared with live bacteria, the main advantage of inactivated probiotics, besides safety, is their outstanding stability [41]. In our study, we found that when stored at room temperature for one month, the viable counts of SeL decreased by more than an order of magnitude. After heat inactivation, HiSeL was stored at room temperature for 12 months without any particle aggregation or property changes, showing good stability. Microbially synthesized SeNPs are different from those of physicochemical methods in that microbially synthesized SeNPs generally contain proteins related to the assembly and stability of SeNPs [42,43]. We found the key roles of proteins and lipids in promoting SeNPs biosynthesis through FT-IR spectroscopy. However, which proteins and lipids play key roles in sodium selenite reduction and SeNPs synthesis remain to be investigated.

The multi-epitope vaccine used for immunization is a new type of vaccine developed by our laboratory with BLP as the carrier and the *C. perfringens* epitope group as the antigen display [25]. It has been shown to induce an immune response in mice with just one subcutaneous injection. However, this vaccine still has the inherent disadvantage of multi-epitope vaccines: poor immune effect. We speculate that using HiSeL as an adjuvant can improve the immune effect and that HiSeL has the potential to be an effective immune enhancer for various vaccine types. Antibody levels produced by B cells are a direct indicator of vaccine protection, and higher levels of vaccine efficacy are associated with higher antibody levels [44]. Our results indicate that HiSeL elicits serum IgG and fecal IgA antibodies more rapidly than aluminum adjuvant, suggesting a potential advantage for vaccine strategies, requiring quick immunity. IgG2a and IgG1 antibodies are markers for Th1 and Th2 lymphocytes, respectively, and the ratio of IgG1/IgG2a reflect the tendency of immune response [45]. We found that HiSeL promoted the production of IgG1 and IgG2a at the same time, with a ratio of 0.68 < 1, which was higher than that of the Alum adjuvant (ratio of 0.5).

Furthermore, compared to the Vac group, mice in the Vac + HiSeL group exhibited elevated levels of Th1 cytokine IFN-γ, as well as Th2 cytokines (IL-4 and IL-5). We also observed increased expression of regulatory cytokines, including IL-10 and TGF-β, which are pivotal for maintaining immune homeostasis and facilitating IgA production. Notably, the expression of the pro-inflammatory cytokine TNF-α was reduced, suggesting a controlled inflammatory profile rather than excessive inflammation. Collectively, these cytokine patterns suggest that HiSeL may elicit a mixed Th1/Th2 immune response instead of a strictly polarized phenotype. This integrated profile, reflecting both humoral and cellular immunity, is consistent with our previous findings [19].

In our study, subcutaneous immunization with the HiSeL-adjuvanted multi-epitope vaccine could induce the rapid secretion of SIgA antibodies. Currently, most approved vaccines are administered by systemic (e.g., intramuscular and subcutaneous) routes. However, the parenteral route of immunization is generally considered ineffective at generating mucosal immune responses. There have been numerous studies demonstrating that LAB and selenium can both induce IgA production [46,47,48], but this is usually achieved through oral immunization. Notably, this study demonstrated that subcutaneous immunization with HiSeL significantly increased the SIgA antibody titer in the feces in 21 and 28 dpi, and significantly increased the total SIgA content in the jejunal mucus (*p* < 0.001) by subcutaneous immunization. Furthermore, we detected cytokines related to SIgA production such as IL-4, IL-5, IL-10 and TGF-β, and observed that HiSeL significantly upregulated the expression of these genes. These results are also consistent with our RNA-seq analysis, which showed that the “intestinal immune network for IgA production” pathway was involved in the immune response. Previous studies have reported that parenteral BCG vaccination can induce mucosal immune-related changes, and that the gut microbiota-mediated pathway triggered by parenteral vaccines may promote immune responses in distal mucosal tissues. This aligns with the observed induction of SIgA by HiSeL immunization in this study, suggesting potential mechanistic similarities. However, further research is needed to validate this hypothesis.

SIgA represents the predominant immunoglobulin type in mucosal secretions and serves as a critical first-line defense against infections [19]. Unlike systemic IgG, which primarily functions in blood and tissues, SIgA provides localized protection at mucosal surfaces—the primary entry sites for most pathogens (. Notably, mucosal IgA has been shown to dominate the early neutralizing antibody response to SARS-CoV-2, with SIgA demonstrating superior neutralization potency against the authentic virus compared to IgG. Our findings of enhanced protection despite comparable IgG levels further highlight the potential dominance of mucosal immunity in mediating the protective effects observed with HiSeL.

Only 6 days after immunization, the immune response induced by Vac + HiSeL provided 100% protection against a lethal dose of *C. perfringens* challenge and reduced the weight loss and ileal damage caused by the challenge. A similar phenomenon was also observed in our previous study. We speculate that this positive result is possibly attributable to the rapid production of antibodies.

We observed that HiSeL effectively relieved vaccine-induced increases in heat shock-related genes *Hsp70* and *Hsp90*. It is well known that the body’s stress reaction leads to a decline in immune function. A previous study also found that selenium-rich probiotics reduced the expression of heat shock-related genes (*Hsp90* and *Hsp70*) [49]. Selenium could have mitigated the decline in immune function triggered by lead-induced expression of cytokines and *Hsps* (27, 40, 60, 70, and 90) in chicken neutrophils [50]. However, in our research, the expression of *StAR* and *P450scc* in the Vac and Vac + HiSeL groups was significantly lower than that in the control group. We speculate that this may be due to a compensatory phase in which the body returns to a homeostatic state. A similar phenomenon was observed in the study of Beckmann et al., who found that *GR* and *P450scc* were subsequently downregulated at 3–5 days [51].

Next, we further investigated the primary mechanism of HiSeL by RNA-sequencing. Through GO and KEGG analyses, we found that HiSeL broadly enriched immune-related pathways, which is consistent with the excellent immune indicators previously induced by HiSeL. The top KEGG pathway was the “Hematopoietic cell lineage”. Hematopoietic cells are well-known progenitors of many blood cells that can differentiate into either lymphoid or thymic lineages. The former includes B cells, T cells, and natural killer cells, while the latter includes red blood cells, granulocytes, megakaryocytes, macrophages, and monocytes. All of these are closely involved in various processes of the immune response. Another top-ranked KEGG pathway was the “Intestinal immune network for IgA production”. This was expected, as HiSeL demonstrated outstanding SIgA production capacity in inducing antibodies and cytokines. Two other significant KEGG pathways, the “TGF-β signaling pathway” and “NF-κB signaling pathway”, were identified in the present study. A previous study found that selenium-enriched *B. subtilis* was recognized by the TLR2 receptor in the ileal mucous membrane, which activated the TLR2 MyD88–NF-κB signaling pathway [51]. The critical role of non-classical NF-κB in IgA class switching has been demonstrated in multiple mouse models [52]. However, to our knowledge, no study has investigated the association between selenium-rich probiotics and the TGF-β signaling pathway. TGF-β is a multifunctional growth factor that regulates and maintains immune system balance [53]. It has been reported that TGF-β signaling regulates B cell responsiveness to antigen, IgA production, and IgA class switch recombination on SMAD-dependent pathways [54]. Furthermore, TGF-β signaling plays a role in the early development of Treg cells, and 3–5-day-old mice lacking the TGF-β type I receptor (TβRI) exhibit defects in IgA production [55]. To further explore whether the TGF-β and NF-κB signaling pathways activated by HiSeL were related to IgA production, we constructed a PPI network using DEGs from these three pathways. Results showed that the TGF-β and NF-κB pathway proteins are closely related to the intestinal immune network for IgA production. In addition, we identified key hub proteins, namely TGF-β1, MAP3k14, and CXCL12. MAP3K14 (also known as NF-κB-inducing kinase), which play a crucial role in antigen activation of cells and both innate and adaptive immune responses [56]. It regulates the non-canonical NF-κB pathway, influencing the development of lymphoid organs, activation of dendritic cells, and maturation and survival of B cells [57]. Cxcl12, a chemokine, facilitates the homing of immune cells during the immune response, particularly in the germinal center reaction. Additionally, Cxcl12 can induce NF-κB non-canonical pathway activation via an IKKα-dependent mechanism.

Since Peyer’s patches are secondary lymphoid organs located in the small intestine, they are important for inducing antigen-specific intestinal immune responses [58,59]. We used RT-qPCR to detect the expression of the hub genes (*TGF-β1*, *Map3k14* and *Cxcl12*) and other important genes (*J Chain*, *IκB* and *PIgR*) in Peyer’s patches and spleen, and the results confirmed the RNA-seq findings. Among them, the mRNA levels of IκB exhibited an inverse relationship with the activation process of the classical NF-κB signaling pathway. Activation of the classic NF-κB signaling pathway promotes the phosphorylation and degradation of the IκB protein, which leads to the release and nuclear translocation of the NF-κB dimer. In summary, our data suggests that HiSeL-enhanced IgA production is associated with the activation of TGF-β and non-canonical NF-κB signaling pathways.

Effective adjuvants are required to enable sufficient protection from multi-epitope vaccines, and our data confirms HiSeL as a potent candidate. Nevertheless, we acknowledge limitations regarding the mechanistic dissection of this composite. FTIR spectra suggested that organic residues (proteins and lipids) are integral to the SeNPs, yet their specific profiles were not determined. Moreover, the independent roles of the SeNPs and the bacterial carrier were not isolated in this study. Future investigations should aim to dissect these components; such comprehensive analysis will be crucial for distinguishing the specific immunomodulatory effects of the selenium from those of other bacterial constituents. While the *i.p.* challenge model demonstrates the vaccine’s ability to induce neutralizing antibodies that prevent toxin-mediated lethality, it does not mimic the complex gut colonization and intestinal pathology of necrotic enteritis in chickens. Finally, while RNA-seq and qPCR analyses provided robust evidence for the upregulation of TGF-β and NF-κB pathways, we acknowledge that this study established a correlation rather than direct causation. Future studies utilizing specific pathway inhibitors or knockout mouse and chicken models are necessary to functionally verify that blocking these pathways abolishes the adjuvant effect of HiSeL.

## 5. Conclusions

In conclusion, we found that due to heat inactivation treatment, selenium-enriched *Lactobacillus* exhibit perfect stability. Subcutaneous immunization with the HiSeL-adjuvanted multi-epitope vaccine elicits robust protective immunity, characterized by elevated titers of high-affinity and toxin-neutralizing IgG, a balanced IgG subclass profile, and the upregulation of immunoregulatory cytokines. Crucially, this regimen confers complete protection against *C. perfringens* challenge while significantly mitigating the systemic stress response. And more importantly, HiSeL induced excellent IgA responses, which are essential for immune protection at mucosal sites. Transcriptome profiling suggests that this enhanced mucosal immunity is associated with the activation of TGF-β and NF-κB signaling pathways. Collectively, our study provides a proof-of-concept for HiSeL as a novel adjuvant candidate. These findings warrant further evaluation in poultry to validate its translational potential for preventing necrotic enteritis.

## Data Availability

The original data presented in the study are openly available in RNA-seq data SRA database under project number: PRJNA1272497.

## Supplementary Materials

The following supporting information can be downloaded at https://www.mdpi.com/article/10.3390/microorganisms14010180/s1; Figure S1: Heat map of selenoproteins and antioxidant genes expression; Figure S2: The expression of J chain, IκB, and pIgR, comparison between RT-qPCR’s 2-ΔΔCt (bars for spleens and blue broken lines for Peyer’s patches) and RNA-seq’s TPM (red broken lines) is presented. The significant labeling in the figure (*: *p* < 0.05, **: *p* < 0.01) based on RT-qPCR test results of spleens; Table S1: PCR primers sequences. References [60,61,62,63,64,65,66,67] are cited in the supplementary materials.
