# Supplementary material for: Heat-Inactivated Selenium Nanoparticle-Enriched Lactobacillus Enhance Mucosal IgA Responses and Systemic Responses of Clostridium perfringens Multi-Epitope Vaccine Correlated with TGF-β and NF-κB Pathways in Mice
Source: Microorganisms. 2026 Jan 14;14(1):180. doi: 10.3390/microorganisms14010180 (PMC12844021; doi:10.3390/microorganisms14010180)
Supplement: Supplementary file 1 [file microorganisms-14-00180-s001.zip › microorganisms-4043478-supplementary.pdf]

Supplementary Material

Supplementary Table S1. PCR primers sequences

Gene	Primer name	Primer sequence (5'-3')	Reference
β-actin	β-actinF	TGACAGGATGCAGAAGGAGA	Self-designed
	β-actinR	GCTGGAAGGTGGACAGTGAG	
IL-4	IL-4F	CGGCACATCTACAGACACCAC	Self-designed
	IL-4R	CTTCATGCACAGAACAGGTC	
IL-10	IL-10F	CGCAGCTCTAGGAGCATGTG	Self-designed
	IL-10R	GCTCTTACTGACTGGCATGAG	
IFN-γ	IFN-γF	AGACAATCAGGCCATCAGCA	Self-designed
	IFN-γR	TGGACCTGTGGGTTGTTGAC	
IL-1β	IL-1βF	TCTCCAGCCAGTCTTCATTGT	Self-designed
	IL-1βR	GCCATCAGCCTCAAATAACAG	
TNF-α	TNF-αF	ACCAGCCAGGAGAGAGACAAG	Self-designed
	TNF-αR	AGCGTGTGAGAGGGAGAGAGT	
Star	StarF	CTTGGCTGCTCAGTATTGAC	[1]
	StarR	TGGTGGACAGTCCTTAACAC	
P450scc	P450sccF	CGCATCAAGCAGCAAAATTC	[2]
	P450sccR	ATGCGCTCCCCAAATATAAC	
HSP70	HSP70F	ATGGACAAGGCGCAGATCC	[3]

	HSP70R	CTCCGACTTGTCCCCCAT	
Hsp90	Hsp90F	CCTGGGAACCATTGCTAAGTCT	4
	Hsp90R	GCCCGATCATGGAGATGTCT	
TGF-β1	TGF-β1F	CCACCTGCAAGACCATCGAC	Self-designed
	TGF-β1R	CTGGCGAGCCTTAGTTTGGAC	
Cxcl12	Cxcl12F	TGCATCAGTGACGGTAAACCA	5
	Cxcl12R	TTCTTCAGCCGTGCAACAATC	
Map3k14	Map3k14F	TGCGACTTTGGCCACGCCTT	6
	Map3k14R	GCCATGTGGGTCTCCGTGCC	
J chain	J chainF	AGCGACCATTCTTGCTGACA	Self-designed
	J chainR	TCCTCGGTGGAAGGGATGAT	
IκBα	IκBαF	GAGACCTGGCCTTCCTCAAC	7
	IκBαR	TCTCGGAGCTCAGGATCACA	
pIgR	pIgRF	AGGCAATGACAACATGGGG	8
	pIgRR	ATGTCAGCTTCCTCCTTGG	

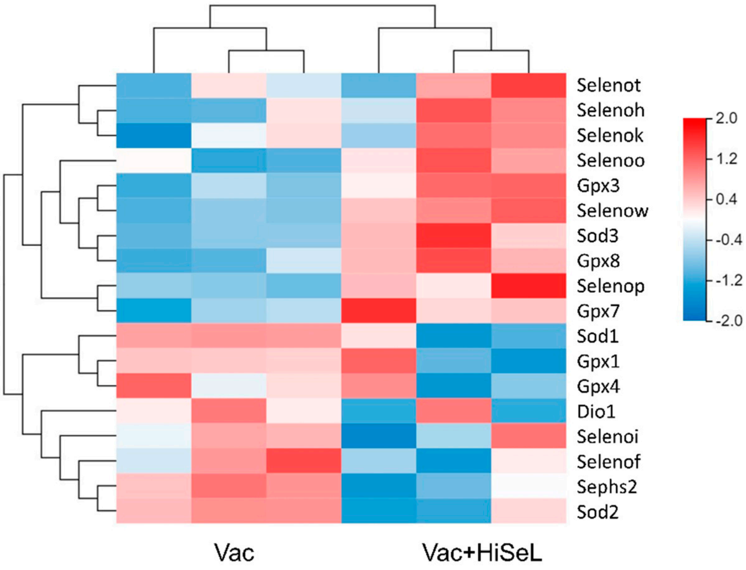

Supplementary Figure S1 Heat map of selenoproteins and antioxidant genes expression.

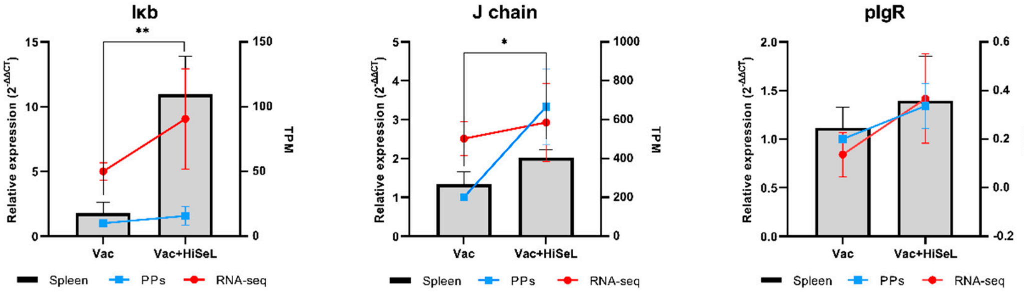

Supplementary Figure S2 The expression of J chain, IκB, and pIgR, comparison between RT-qPCR's 2-ΔΔCt (bars for spleens and blue broken lines for Peyer's patches) and RNA-seq's TPM (red broken lines) is presented. The significant labeling in the figure (*: P < 0.05, **: P < 0.01) based on RT-qPCR test results of spleens.

SUPPLEMENTARY REFERENCES

1. Cheng, X. B. *et al.* Characterizing the neuroendocrine and ovarian defects of androgen receptor-knockout female mice. *American Journal of Physiology-Endocrinology and Metabolism* **305**, E717–E726 (2013).

2. Xu, B. *et al.* Dax-1 and Steroid Receptor RNA Activator (SRA) Function as Transcriptional Coactivators for Steroidogenic Factor 1 in Steroidogenesis. *Molecular and Cellular Biology* **29**, 1719–1734 (2009).

3. Rossin, F. *et al.* TG2 regulates the heat-shock response by the post-translational modification of HSF1. *EMBO Reports* **19**, e45067 (2018).

4. Casas, S. *et al.* Impairment of the Ubiquitin-Proteasome Pathway Is a Downstream Endoplasmic Reticulum Stress Response Induced by Extracellular Human Islet Amyloid Polypeptide and Contributes to Pancreatic β-Cell Apoptosis. *Diabetes* **56**, 2284–2294 (2007).

5. Patnaik, A. *et al.* Cabozantinib Eradicates Advanced Murine Prostate Cancer by Activating Antitumor Innate Immunity. *Cancer Discovery* **7**, 750–765 (2017).

6. Jin, J. *et al.* The kinase TBK1 controls IgA class switching by negatively regulating noncanonical NF-κB signaling. *Nat Immunol* **13**, 1101–1109 (2012).

7. Yang, C. *et al.* Fecal IgA Levels Are Determined by Strain-Level Differences in Bacteroides ovatus and Are Modifiable by Gut Microbiota Manipulation. *Cell Host & Microbe* **27**, 467-475 (2020).

8. Mountz, J. D., Wu, J., Zhou, T. & Hsu, H. C. Cell death and longevity: implications of Fas-mediated apoptosis in T-cell senescence. *Immunol Rev* **160**, 19–30 (1997).
